# Supplementary material for: Soil acidity and fertility status of surface soils under different land uses in Sayo district of Oromia, western Ethiopia
Source: PLoS One. 2024 Dec 26;19(12):e0316009. doi: 10.1371/journal.pone.0316009 (PMC11670963; doi:10.1371/journal.pone.0316009)
Supplement: Attachment — Submitted filename: Response to Reviewers.docx [file pone.0316009.s003.docx]

**College of Agriculture and Natural Resource
Dambi Dollo University**

**Dambi Dollo City, Ethiopia**

**Dear reviewers and Editor**,

We are thankful for your kind comments on our manuscript entitled “**Soil acidity and fertility status of surface soils under different land uses in Sayo district of Oromia, western Ethiopia**” following our submission for publication in PLOSE ONE journal. We have re-looked at the manuscript and changed it in line with your comments as follows.

We have incorporated your comments on the abstract and revised it to enhance its attractiveness. Based on your suggestions, we revised the introduction, including the statement of the problem by clearly setting the research gap. Also, we added several new sentences to improve the strength of the introduction. There were no changes made to the materials and methods section, as we did not receive any recommendations regarding the use of specific statistical methods. Therefore, we decided to continue with the methods previously employed unless further suggestions are provided. In response to your comments, we revised the results and discussion sections, ensuring the interpretation aligns with our findings and is supported by recent literature. Additionally, we have corrected grammatical and technical errors to ensure consistency throughout the discussion. Finally, we have modified the conclusion part of the manuscript by providing your constructive comments.

For the editor, the majority of your comments were based on the author's instructions, thus we took all necessary steps to improve the manuscript. Overall, what we want to inform you by using this opportunity is that we updated, rephrased and changed more than half of the manuscript. We respectfully request that you consider this modified text for publication.

**Sincerely,**

**Abu Regasa (PhD candidate)**

**Dambi Dollo University**

**On behalf of all authors**
