# Supplementary material for: Soil acidity and fertility status of surface soils under different land uses in Sayo district of Oromia, western Ethiopia
Source: PLoS One. 2024 Dec 26;19(12):e0316009. doi: 10.1371/journal.pone.0316009 (PMC11670963; doi:10.1371/journal.pone.0316009)
Supplement: Attachment — Submitted filename: response to reviewers.docx [file pone.0316009.s004.docx]

**College of Agriculture and Natural Resource
Dambi Dollo University**

**Dambi Dollo City, Ethiopia**

**Dear Reviewers and Editor,**

We would like to express our gratitude for your constructive comments and suggestions on our manuscript entitled *“****Soil Acidity and Fertility Status of Surface Soils under Different Land Uses in Sayo District of Oromia, Western Ethiopia”*** submitted for publication in ***PLOS ONE***. Following your feedback, we have thoroughly revised the manuscript to address the points raised, as detailed below:

**Journal Requirements and References**:

We have addressed the comment regarding retracted articles by removing them from our references. In addition, we have replaced the preprint reference: ***Regasa A. Conversion in Land Use Change Soil Physiochemical Properties in the Highland of Western Ethiopia, 29 September 2023, PREPRINT (Version 1) available at Research Square [***[***https://doi.org/10.21203/rs.3.rs-3371625/v1***](https://doi.org/10.21203/rs.3.rs-3371625/v1)***]. 2023*** with alternative and more suitable citations. We have included one unpublished dataset titled ***“Kellem Wolega Zone Agriculture and Natural Resource Office. Description of Kellem Wolega Zone Dambi Dollo City, Oromia; 2022****”* to provide local and contextual data relevant to our analysis.

**Supplementary Materials**: In line with your suggestion, we have combined the supplementary materials into a single table (***S2 Table***). This ensures clarity and better alignment with journal requirements.

**Statistical Reporting**: We have updated Tables 1, 2, and 3 by including F-values and significance levels, enhancing the statistical robustness and transparency of our findings.

**Editorial Issues**: All highlighted editorial issues have been addressed. For instance, we revised the final paragraph of page 3 to clearly articulate the specific objectives of the study, improving clarity and coherence.

**Site Selection and Soil Sampling**: We have expanded the discussion of site selection and soil sampling methods by providing detailed descriptions of all land use types, including natural forest land. This addition ensures a more comprehensive understanding of the study context.

We respectfully request that the revised manuscript be considered for publication. We are confident that these revisions address the concerns raised and contribute to improving the quality of the manuscript.

Thank you for your time and valuable input, and we look forward to your decision.

**Sincerely,**

**Abu Regasa (PhD candidate)**

**Dambi Dollo University**

**On behalf of all authors**
